# Measurements Methods for the Development of MicroRNA-Based Tests for Cancer Diagnosis

**DOI:** 10.3390/ijms22031176

**Published:** 2021-01-25

**Authors:** Francesca Precazzini, Simone Detassis, Andrea Selenito Imperatori, Michela Alessandra Denti, Paola Campomenosi

**Affiliations:** 1 Dipartimento di Biologia Cellulare, Computazionale e Integrata (CIBIO), Università degli Studi di Trento, Via Sommarive 9, 38123 Trento, Italy; f.precazzini@unitn.it; 2 OPTOI Srl, Via Vienna 8, 38121 Trento, Italy; simone.detassis@optoi.com; 3 Dipartimento di Medicina e Chirurgia (DMC), Università degli Studi dell’Insubria, Via Guicciardini 9, 21100 Varese, Italy; andrea.imperatori@uninsubria.it; 4 Dipartimento di Biotecnologie e Scienze della Vita (DBSV), Università degli Studi dell’Insubria, Via Dunant 3, 21100 Varese, Italy

**Keywords:** microRNA, biomarker, biofluids, methods for miRNA detection, diagnostic test

## Abstract

Studies investigating microRNAs as potential biomarkers for cancer, immune-related diseases, or cardiac pathogenic diseases, among others, have exponentially increased in the last years. In particular, altered expression of specific miRNAs correlates with the occurrence of several diseases, making these molecules potential molecular tools for non-invasive diagnosis, prognosis, and response to therapy. Nonetheless, microRNAs are not in clinical use yet, due to inconsistencies in the literature regarding the specific miRNAs identified as biomarkers for a specific disease, which in turn can be attributed to several reasons, including lack of assay standardization and reproducibility. Technological limitations in circulating microRNAs measurement have been, to date, the biggest challenge for using these molecules in clinical settings. In this review we will discuss pre-analytical, analytical, and post-analytical challenges to address the potential technical biases and patient-related parameters that can have an influence and should be improved to translate miRNA biomarkers to the clinical stage. Moreover, we will describe the currently available methods for circulating miRNA expression profiling and measurement, underlining their advantages and potential pitfalls.

## 1. Introduction

Although about 90% of the eukaryotic genome is transcribed into RNA, only 1–2% of these transcripts encode for functional proteins [1,2]. This indicates that most of the transcripts are transcribed as non-coding RNAs (ncRNAs). ncRNAs have an important role in the transcription of protein-coding genes, maturation of the transcripts and their translation into functional proteins [3].

### 1.1. Types of Non-Coding RNAs

The majority of ncRNAs belong to the group of long ncRNAs (lncRNAs) that are usually >200 nt in length. Many of the lncRNAs are subject to splicing, polyadenylation and other post-transcriptional modifications, and can also be classified according to their proximity to protein-coding genes [4]. ncRNAs with length <200 nt are called small ncRNAs and include the most common transfer RNAs (tRNAs), 5S ribosomal RNA (5S rRNA), but also small interfering RNAs (siRNAs), piwi-interacting RNAs (piRNAs), small nuclear RNAs (snRNAs), small nucleolar RNAs, YRNAs and microRNAs (miRNAs) [5,6].

siRNAs are 20–24 nt-long molecules deriving from long linear dsRNAs processed by Dicer, that direct silencing when loaded onto the RNA-induced silencing (RISC) complex. During RNAi they mediate post-transcriptional silencing by interfering with the expression of targets with complementary nucleotide sequences [7].

piRNAs (24–31 nt) are characterized by a uridine at the 5′end and a 2′-O-methyl modification at the 3′ end. They can form complexes with Piwi proteins of the Argonaute family, that represent a key player in the RNA interference process. Moreover, Piwi/piRNA complexes in mammals and flies are related to the control of transposable elements during germline development [8].

snRNAs (~150 nt) are often found within the Cajal bodies in eukaryotic cells. They are transcribed by either RNA pol II or III. Their primary function consists in the processing of pre-mRNAs in the nucleus. They have also been shown to act in the regulation of transcription factors and in the maintenance of telomeres [9].

Small nucleolar RNAs (snoRNAs) are approximately 60 to 170 nt in length and mainly have a role in the post-transcriptional modification of uridines (pseudouridylation) or the 2′-*O*-methylation of rRNAs and other transcripts [10].

YRNAs (100 ± 20 nt) fold into characteristic stem-loop secondary structures and are characterized by a single bulged cytosine. They are components of the Ro60 ribonucleoprotein particle and have an active role in DNA replication thanks to the interaction with initiation proteins including the origin recognition complex and chromatin. Humans have four YRNAs named hY1, hY3, hY4, and hY5 [11,12].

MicroRNA (miRNAs) are short, single-stranded ncRNAs (approximately 22 nt in length) that inhibit gene expression. They were first discovered in 1993 in *Caenorhabditis elegans* [13]. Since then, miRNAs have been found in vertebrates and invertebrates, and some of them are highly conserved [14]. Interestingly, miRNAs were also reported in viruses [15].

Among the described non-coding RNAs, miRNAs have been extensively studied and are the best characterized not only from the biological point of view, but also regarding their application for the diagnosis of human disease, including cancer [16,17,18], and their application for diagnosis and prognosis assessment reviewed in [19,20]. 

Nevertheless, there are technical and biological challenges in their measurement, especially in body fluids. This prompted the scientific community to develop new technologies or adapt classical methods for their quantification. This review will focus on the technologies that have been or are currently under development for miRNA quantification in body fluids, focusing in particular in miRNA measurement applied to the cancer field.

### 1.2. Focusing on miRNAs

Generally, mammalian miRNAs are encoded in the genome and transcribed as initial longer transcripts called pri-miRNAs by RNA Polymerase II. The cleavage of pri-miRNAs by the DROSHA-DGCR8 complex leads to the production of precursor miRNAs (pre-miRNAs) that are exported into the cytoplasm in a process mediated by exportin 5 (*EXP5*). The RNase III-like DICER cleaves the stem-loop of the pri-miRNA to generate a ds-miRNA, that is then incorporated into the RNA-induced silencing complex (RISC) through the binding with an Argonaute family member. One strand of the ds-miRNA is maintained in the RISC complex while the other strand (passenger strand) is degraded [21]. MiRNAs function mainly by binding to the 3′UTR of the target mRNA thanks to sequence complementarity. In case of perfect pairing mRNA degradation will occur, while imperfect pairing results in mRNA sequestration and translation inhibition [22]. Interactions of miRNAs with other regions, including the 5′UTR, coding sequence, or gene promoters, have also been reported in the literature [23]. The first evidence in support of the important role of miRNAs in biological processes was the discovery that Dicer1 gene disruption in mice led to early embryonic lethality, with Dicer1-null embryos depleted of stem cells [24].

The levels of expression of specific miRNAs can be differently tuned in tissues and organs. Several miRNAs display a cell- or tissue-specific expression profile: For example, muscle-specific miRNAs, miR-1 and miR-133 in particular, play important roles in muscle cell proliferation and differentiation [25].

Regulation of miRNA expression has been shown to be affected by epigenetic changes like histone modification or DNA methylation and by proteins involved in miRNAs maturation [26] such as the RNA helicases p68 and p72, that play a critical role in the post-transcriptional regulation of numerous miRNAs in response to cellular signals such as TGF-β stimulation, estrogen stimulation or DNA damage [27].

Dysfunction or dysregulation of miRNAs were reported in several types of diseases: Cancer [16,17,18], viral diseases [28,29], immune-related diseases [30,31], cardiac pathogenic conditions [32], and neurodegenerative diseases like Alzheimer’s [33] or Parkinson’s [34].

### 1.3. miRNAs in Biological Fluids

miRNAs do not act only intracellularly, but they can mediate cell-cell communication by means of their incorporation into extracellular vesicles (EVs), that are secreted by most cell types in the extracellular space and in different biological fluids. Since EVs contain different bioactive molecules like proteins and miRNAs, they can affect molecular pathways and protein expression of recipient cells. Thus, they represent an important aspect of intercellular communication and gene regulation [35]. Circulating miRNAs (c-miRNAs) can also be complexed with proteins like argonaute protein 2 (Ago2) [36]. Other proteins were found to bind extracellular miRNAs, including high-density lipoprotein (HDL) [37] and nucleophosmin 1 (NPM1) [38]. Association with proteins increases resistance to degradation by ribonucleases and hence results in a higher stability of miRNAs in the extracellular environment compared to other biological molecules [39,40].

miRNAs can be found in different body fluids such as saliva [41], blood and its derivatives serum and plasma [42], breast milk [43] and urine, among others [44]. Cell-free miRNAs from plasma and serum are the most commonly studied circulating miRNA biomarkers, but other body fluid samples such as urine and saliva are sources of c-miRNAs [45]. miRNAs present in the urine tract derive from detached cells or EVs released from kidney or urinary tract cells and could act as a source of biomarkers for several disorders. Deep Sequencing profiling of urine miRNAs was performed by Ben-Dov et al., who showed that time of voids does not seem a variable affecting miRNA levels, while gender and fractionation of the sample (EVs vs. sediment cells) may be important aspects to consider when dealing with urine miRNA biomarker studies [46].

### 1.4. miRNAs as Diagnostic and Prognostic Biomarkers

**Figure 1:**
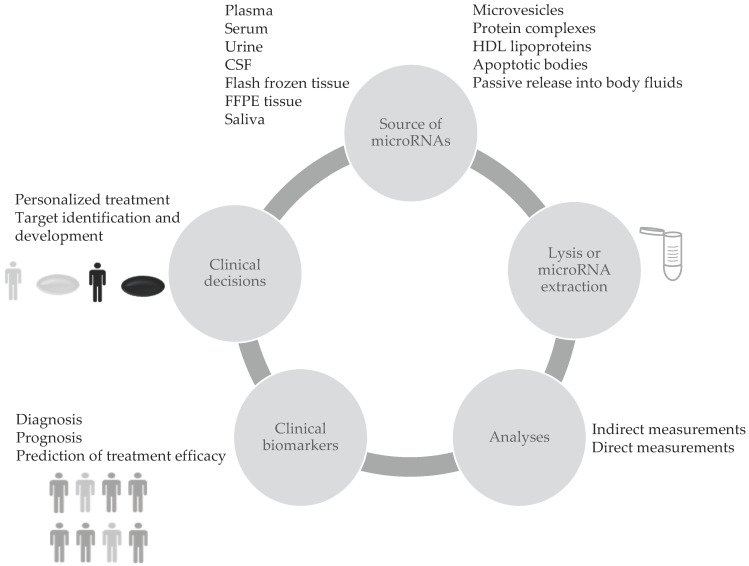
miRNAs are involved in all cellular processes and dysregulated in disease, potentially representing optimal biomarkers. They can be purified from different tissues or liquid biopsies and then measured with the different technologies described in the text. Differential expression of miRNAs may indicate an increased risk of disease or have a diagnostic or prognostic value, leading to clinical decisions.

miRNA molecules are resistant to extreme temperatures, pH, and formalin-fixed paraffin-embedding (FFPE) processing. Storing FFPE tissue samples is more economical than storing frozen samples and histological properties can be preserved. Kakimoto and collaborators showed that miRNAs are stably detected in postmortem tissue samples and that qPCR analysis can be performed after extended formalin fixation [47]. Also miRNA deep sequencing has been performed using FFPE samples, and good overall correlation with the results obtained by analyzing matched frozen samples has been reported [48,49].

c-miRNAs can be considered as valuable biomarkers since they have some advantages such as high stability, resistance to ribonucleases and to severe physicochemical conditions in body fluids, increasing the feasibility of their use in clinical applications. Differential expression of c-miRNAs showed big potential for cancer screening. Thus, these small RNA molecules may function as clinical biomarkers to identify the presence of tumors, to select treatment strategies and to predict outcomes as explained in Figure 1 (for other reviews see [50,51]). Composition and levels of extracellular miRNAs can vary as a consequence of diseases or other conditions [52]. Several reports show that miRNAs are sensitive to physiological or pathological changes, specific to the pathology of interest, and they represent a reliable indicator of the presence of disease before clinical symptoms appear [53]. Moreover miRNAs have shown great potential as diagnostic biomarkers for neurodegenerative disorders [54,55,56], cardiovascular diseases [57], endometriosis [58], cancer (see [59,60,61,62,63,64] among others), and several other diseases.

### 1.5. miRNAs Alterations and Cancer

Regarding more specifically miRNAs and cancer, there are several works showing that microRNAs dysregulation affects cancer biology, being involved in all the hallmarks of cancer as both tumor suppressors and onco-miRs [50]. miRNAs are involved in different aspects of tumorigenesis, sustaining the proliferative signaling, enabling replicative immortality or reprogramming energy metabolism, among others. Decrease of miR-545 was found in colorectal cancer (CRC) in comparison to normal tissues and its over-expression resulted in lower cell proliferation capacity [65]. The miR-130b-301b cluster, a regulator of the senescence mechanism, is hypermethylated in prostate cells; its ectopic expression can restore senescence mechanisms, reducing the malignant phenotype of prostate cancer cells [66]. Dysregulation of cell metabolism is another hallmark of cancer that has been shown to be influenced by miRNAs. Reactive oxygen species (ROS) are involved in different physiological cellular functions and are often increased in cancer cells. High levels of ROS may oxidize DNA or RNA, leading to lesions in the genome which may contribute to cancer progression [67,68]. miR-1 was found to be down-regulated in CRC cell lines compared to normal colon epithelial cells. Restoration of its expression decreased cancer cell proliferation by diminishing glucose uptake, production of lactate and aerobic glycolysis in vitro, impacting SMAD3 pathway and targeting HIF-1α [69].

At the beginning of this century, several groups found that expression patterns of miRNAs could be used to discriminate tumor subtypes. Using a bead-based flow cytometric miRNA expression profiling method Lu and colleagues developed miRNA fingerprints to better classify and distinguish tumor origin as well as to understand the degree of their differentiation [70]. Indeed it was demonstrated that miRNA signature could be used to differentiate lung adenocarcinoma from squamous cell carcinoma tissues and for diagnostic and prognostic purposes [17]. In addition, the combination of miR-21 and miR-205 was found to be able to distinguish lung adenocarcinoma from squamous cell carcinoma [71]. However, the ability of miR-205 to discriminate ADC versus SCC lung cancer histotypes was only partially confirmed in a subsequent study by Del Vescovo et al. [72]. In muscle-invasive bladder cancer (MIBC) a signature of sixty three miRNAs that allowed to discriminate basal and luminal subtypes was validated. This study also confirmed that patients with basal subtype tumors had shorter overall survival [73].

In 2017 Zhang et al published a study in which miRNAs and mRNAs microarray analyses were performed on the same samples. A miRNA-mRNA regulatory network was obtained in order to identify miRNA candidates associated with lung cancer through integration of gene expression and miRNA-target prediction data. Twenty-eight miRNAs associated with lung cancer were identified by microarray, TCGA and miRNA-mRNA network analysis, obtaining a preliminary miRNA signature which may become a promising biomarker for the early screening of high-risk populations and early diagnosis of lung cancer [74]. 

In 2019 miR-223 was validated as a reproducible, effective serum biomarker of early-stage non-small-cell lung cancer using ddPCR technology [75]. A recent study from Detassis and colleagues demonstrated that miR375-3p distinguishes low-grade neuroendocrine from non-neuroendocrine lung tumors in FFPE samples [76].

Evidence supports a correlation between expression of specific miRNAs in tumor tissue and their level in biofluids. In 2019 Mjelle et al performed small RNA profiling and gene expression arrays of tumor, peritumoral tissues, circulating exosomes and serum from the same hepatocellular carcinoma patients and circulating exosomes and serum from non-cancer controls and found a correlation between miRNAs dysregulated in tumor samples and levels of circulating miRNAs, in particular those contained in exosomes [77]. In a study by Xue et al., exosomal miRNA plasma profiling was performed from six lung adenocarcinoma patients before and after surgery. miR-484 was found significantly increased in plasma of lung adenocarcinoma patients compared to controls, but it decreased post-surgery [78].

However, in some instances, the relation between circulating miRNAs and miRNA expression in tumor tissue may not be so straightforward. For example, miR-223 is expressed in hematopoietic cells and secreted in exosomes, influencing invasivity and other behaviors of tumor recipient cells [79]. 

The results of these and other studies support the value of miRNA as non-invasive diagnostic biomarkers.

## 2. Technical Limitations in Measurement of c-miRNAs as Biomarkers

If biological complexity is difficult to capture and thus difficult to exploit, there are also technical limitations in c-miRNAs measurement that make it difficult to develop a diagnostic or prognostic test directly translatable in clinical settings. In fact, despite the growing interest of the scientific community, proved by the enormous amount of articles published in recent years (a search for “circulating microRNAs AND cancer biomarker” on PubMed, performed in September 2020, yielded 123 entries in 2012, 189 in 2013, 254 in 2014, 335 in 2015, 329 in 2016, 338 in 2017, 356 in 2018, and 310 in 2019) only two miRNA based diagnostic tests (Biomild, Cosmos II) have reached phase 4 in clinical trials [80]. The main reasons for the limited clinical application may be classified into three categories: (1) Pre-analytical, (2) analytical, and (3) post-analytical factors. These factors affect measurement accuracy and reproducibility independently of its clinical application. 

### 2.1. Pre-Analytical Factors

Although it is widely accepted that c-miRNAs levels reflect physiological and pathological changes, it is apparent that their levels may be influenced by several factors which ultimately affect the assay results. Indeed, it has been suggested that in plasma, smoking status [81], diet [82], active exercise [83] and-in mice sera-circadian rhythm [84] can affect the total levels of c-miRNAs. On the other hand, gender, menstrual cycle, and food intake do not affect total c-miRNAs levels in plasma and serum [45,85]. Inter-individual variability should also be taken into account considering that the concentration of individual miRNAs in plasma ranges from about 9000 to 130,000 copies per μL [86]. The presence of comorbidities is an important issue and a confounding factor [87]. Patient’s condition and lifestyle are a component of variability that may be reduced by applying standardized clinical protocols, an accurate description of comorbidities and more rigorous and transparent criteria for scientific publications, all aspects that can increase reproducibility. General confounding factors are (a) hemolysis [88]; hemolyzed samples could be identified and eventually removed from the analysis by comparing miRNAs known to be enriched in erythrocytes like miR-451 or miR-144 with a miRNA found to be unaffected by hemolysis [89], (b) time from sampling to processing [90], (c) the type of sample (plasma, serum or other biofluids), and (d) if plasma, the choice of the anti-coagulant and centrifugation conditions [21,91,92]. Not only c-miRNAs are affected by sample preparation and storage. Tissue samples may be generally processed and stored as FFPE or as flash-frozen. Formalin fixation may introduce some biases in the stability of miRNAs depending on their GC content [93] and the concordance between the FFPE and flash-frozen samples is lost during time (years) creating discrepancies in analysis done from one type of preparation or the other [48,94].

### 2.2. Analytical Factors

Common analytical methods used for measurement of c-miRNAs comprise two/three main steps: (a) RNA extraction; (b) reverse transcription; (c) miRNA measurement. Several extraction kits are available that have shown different efficiencies in small RNAs recovery [95]. The guanidium thiocyanate plus phenol-chloroform RNA extraction suffers from several limitations. Interestingly, Kim and collaborators showed in 2012 that miRNAs with low GC content are extracted via TRIZOL-based protocol in a total RNA concentration-dependent manner. Starting from a lower number of cells some miRNAs were retained during RNA extraction leading to an apparently differential expression that was actually an artifact, as this differential retention depended on the miRNA’s sequence and structure [96].

Common analytical methods used for measurement of c-miRNAs comprise two/three main steps: (a) RNA extraction; (b) reverse transcription; (c) miRNA measurement. Several extraction kits are available that have shown different efficiencies in small RNAs recovery [93]. The guanidium thiocyanate plus phenol-chloroform RNA extraction suffers from several limitations. Interestingly, Kim and collaborators showed in 2012 that miRNAs with low GC content are extracted via TRIZOL-based protocol in a total RNA concentration-dependent manner. Starting from a lower number of cells some miRNAs were retained during RNA extraction leading to an apparently differential expression that was actually an artifact, as this differential retention depended on the miRNA’s sequence and structure [94].

### 2.3. Post-Analytical Factors

Detection techniques usually rely on relative quantitation, thus a normalization method is necessary. For miRNAs in biofluids the choice of a good reference is problematic and, if incorrect, it may be misleading, indicating artifactual differential expression results [97]. The most common small RNAs used as reference molecules are miR-16, U6 snRNA, and spiked-in cel-miR-39, but there is no general consensus from the scientific community. Some of the most used molecules, e.g., miR-16, have been found as biomarkers of disease by others [98,99,100]; U6 levels were shown to fluctuate across samples [64,97,101,102,103,104,105,106]. Several authors have concluded that a universal endogenous control is unlikely to be discovered and a suitable reference should be assessed every time, considering the different biological conditions of the samples, their nature and the disease for which the biomarker is being developed. Other strategies involve the use of a set of reference molecules instead of a single one or normalizations based on mathematics such as quantile normalization, geometric mean normalization, loess cycle, rank-invariant normalization, and weighted normalization [107,108,109]. For qPCR, software has been implemented for the selection of the best reference from a set of genes, such as Normfinder, geNorm, and BestKeeper, all having similar performances [110]. Exogenous molecules (spike-ins) are used to control for the efficiency of preanalytical and analytical phases and can be used for normalization purposes. Finally, calibration curves with synthetic molecules can be used to obtain the number of molecules present in biological samples, possibly preparing the calibrator in the same matrix and following the same processing. 

Table 1 summarizes the technical limitations described in Section 2.3.

## 3. Indirect Methods for miRNA Measurement, Relying on RNA Extraction and Reverse Transcription (RT)

**Figure 2:**
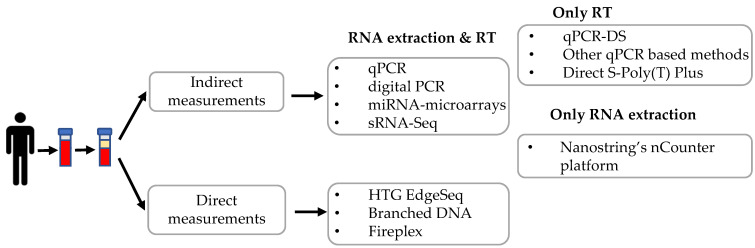
Graphical summary of the different techniques described in this review. RT: Reverse transcription.

Several methods have been applied to quantify c-miRNAs, including quantitative real-time PCR, digital PCR, microarray, and high-throughput small RNA-sequencing. These methods need RNA isolation, in some instances small RNA enrichment, and/or reverse transcription before proceeding to miRNA quantification. A summary of the different techniques described in this review is depicted in Figure 2.

### 3.1. Quantitative PCR (qPCR)

Among the “target amplification-based” methods the most commonly used for the detection of low levels miRNAs is quantitative PCR (qPCR). It is generally considered a highly sensitive method for differential gene expression analysis. Nevertheless, miRNAs are difficult molecules to quantify by PCR for several reasons: The small size prevents optimal primer design; the pre-miRNA is composed of a stable hairpin and the mature miRNA is not discernible from the pre-miRNA, as it corresponds to part of the latter; the presence of different family members differing by just one or few nucleotides; finally, since miRNAs are molecules generally characterized by low abundance in biofluids, their detection can be altered by genomic DNA contamination of RNA samples. Removal of genomic DNA contamination is therefore important before proceeding with the reverse transcription (RT) step. Moreover, small groups of miRNAs can be analyzed with custom-made commercially available plates (e.g., Thermofisher).

**Figure 3:**
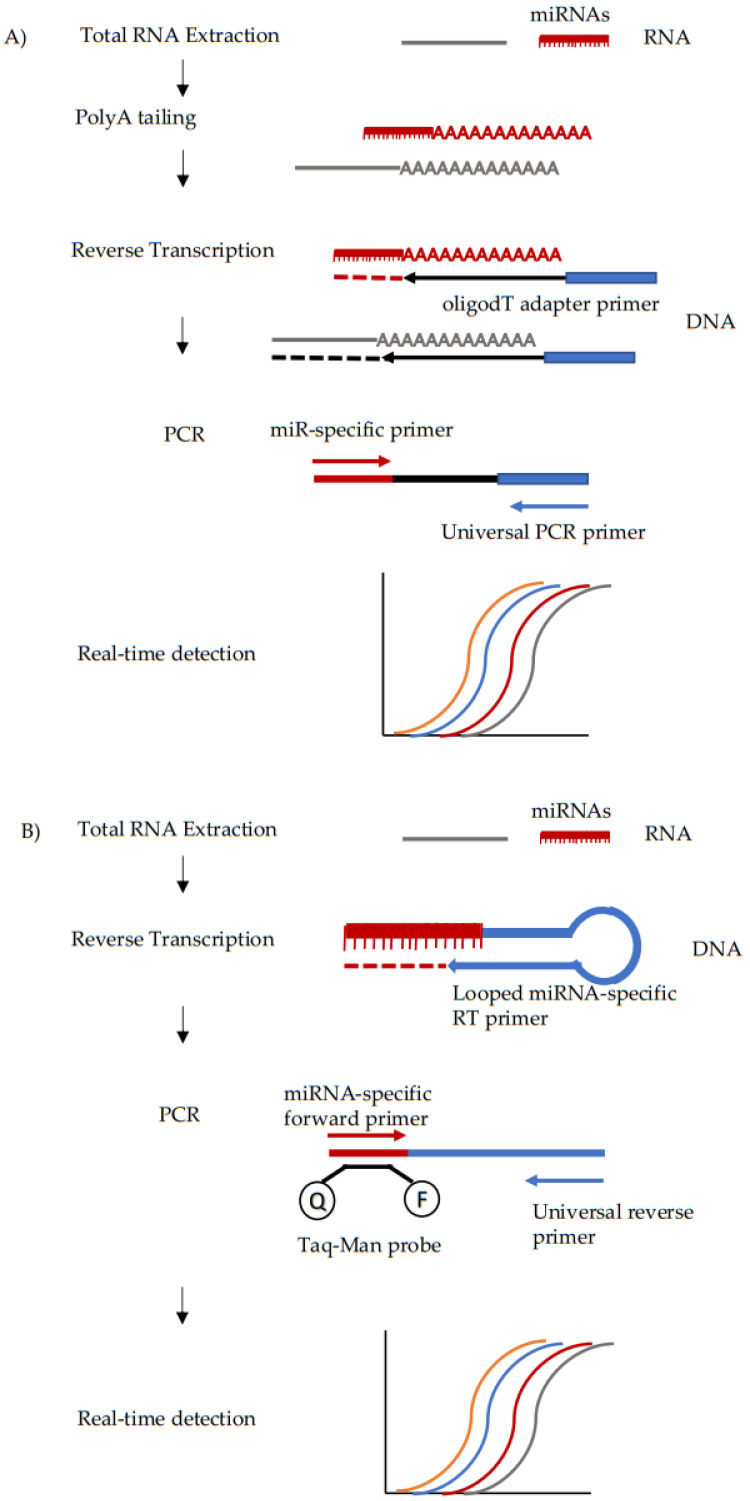
Diagram of quantitative PCR workflow. (**A**) Qiagen and Exiqon methodologies employ PolyA tailing which allows reverse transcription using a universal oligodT primer. This decreases the method’s specificity but improves sensitivity. Real-time detection is enabled by Sybr-green or similar intercalating fluorophores. (**B**) TaqMan RT-qPCR relies on miRNA-specific RT and PCR primers, and therefore has the disadvantage of needing reverse transcription being done for each miRNA separately. This method has high specificity but slightly lower sensitivity than others. Real time detection is based on miRNA-specific Taq-Man probes. Q = quencher; F = Fluorophore.

Two main strategies that combine retrotranscription and amplification are currently adopted for miRNA detection by qPCR. The first (Qiagen, Exiqon) involves the use of poly(A) extension prior to the reverse transcription process for creating cDNA in a single step, overcoming the limitation of short primer design in qPCR. The analysis is performed with SYBR green or other double strand DNA-binding dyes (Figure 3A). Exiqon assays utilize this method, with LNA probes, allowing better hybridization to the target. The second method, TaqMan technology, involves the use of specific stem-loop primers for cDNA production, enhancing specificity of amplification. qPCR is then performed via fluorescent FAM detection (Figure 3B). It has been reported that Exiqon-like RT-qPCR for miRNAs is generally more sensitive than TaqMan technology, at the expense of specificity [111]. Both qPCR methodologies provide a relative quantification hence a normalizer is necessary. As mentioned previously, the choice of the reference gene is critical [97] and, despite the absence of a general consensus, the most common small RNAs used as normalizers for miRNA analysis are U6, miR-16, and spiked-in cel-miR-39. Therefore, the selection of the methodology depends on the application. Although qPCR has some biological as well as technical limitations that restrict its application in clinical settings, it is relatively easy to perform, economical and data analysis is easy [112,113].

### 3.2. Digital PCR (dPCR)

**Figure 4:**
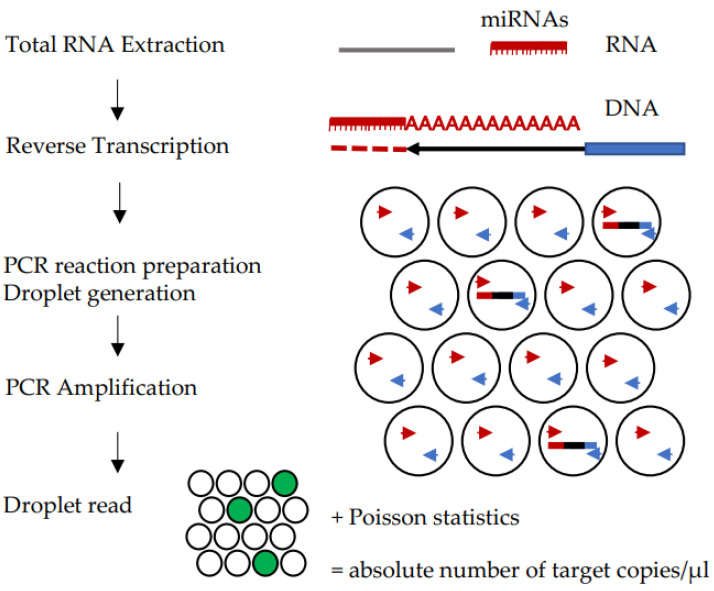
Diagram of droplet digital PCR workflow. Partitioning is the principle underlying quantification. Green colored circles indicate droplets in which target amplification occurred.

More recently, digital PCR (dPCR) became available as a PCR based method capable of absolute quantification. In particular, droplet digital PCR (ddPCR) quantification is based on partitioning of the sample into thousands of micro-reactions of defined volume [114] and quantification is obtained upon completion of PCR, by measuring the fluorescence of each droplet, thus obtaining either positive (fluorescent) or negative (non fluorescent) droplets, that contain or not the nucleic acid of interest (Figure 4): This allows estimation of the number of molecules in the reaction under the assumption of a Poisson distribution, providing the number of copies of target per microliter of reaction, with confidence intervals as result of the analysis [115,116]. Thus, quantification is obtained without the need for reference genes or the construction of a calibration curve, obtaining greater precision compared to qPCR [117,118], also when applied to miRNA quantification [119]. Other advantages of digital PCR over qPCR are the greater precision and sensitivity in detecting low abundance targets [88,91,120,121] and reduced sensitivity to PCR inhibitors [122,123]. Bias in quantification due to pipetting errors can be prevented by performing duplicate reactions. Duplicates also increase the sensitivity in detecting low abundance targets, as data from duplicates can be summed up, increasing the number of measured events. Digital PCR has been shown to have the potential for developing diagnostic and prognostic tests based on miRNA measurements [75,124,125,126,127,128,129,130]. However, like qPCR, digital PCR needs miRNA extraction and reverse transcription and therefore some of the drawbacks of qPCR remain the same. Quantification of exogenous molecules introduced before and between extraction and reverse transcription can be used to check efficiency of these phases. Application of dPCR to miRNA measurement is less widespread than expected: A Pubmed search on “digital PCR AND microRNAs AND biomarkers”, performed on 7th September 2020, yielded about 75 publications regarding miRNA biomarkers for any pathological condition, that decreased to 47 publications describing digital PCR applied for measurement of miRNAs for cancer diagnosis or management. This may be due to the cost of the instrumentation and reagents, to the limited multiplexing capability, the relatively long procedure, the need for trained operators, that may represent limits to its widespread diagnostic applicability.

### 3.3. miRNA Microarrays

**Figure 5:**
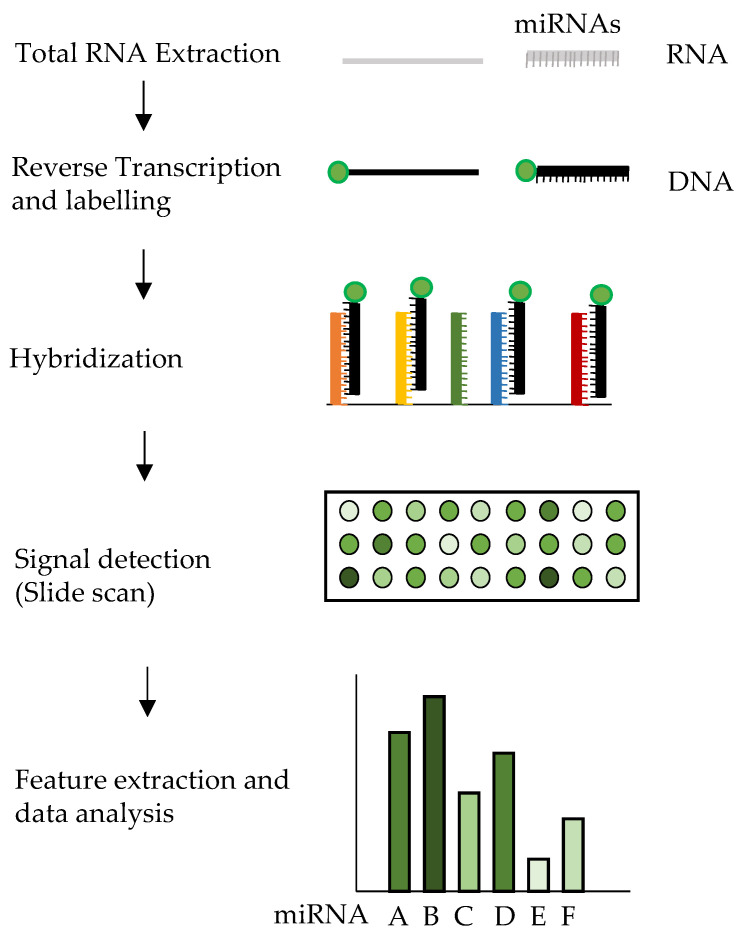
Diagram of miRNAs microarrays workflow. Labelling is usually done by incorporating a fluorophore during the reverse-transcription step. For the sake of simplicity, only one probe is shown per each miRNA type, but several identical probes are present in clusters on the microarray surface, and they bind to identical target miRNAs. Moreover, only 18 probe clusters are depicted on the microarray slide, but a microarray contains thousands of clusters, with up to 20 repetitions of the same cluster in different places of the slide. Wash steps are not shown.

Microarrays represent a high-throughput method used to concurrently detect presence and changes in levels of a wide range of miRNAs in an individual experiment. Microarrays are based on nucleic acid hybridization between target molecules and their corresponding complementary probes. The probes are usually synthesized with a 5′amine-modification that allows for their immobilization on a solid support (e.g., glass slides) by covalent crosslinking. Different fluorescent dyes like Alexa Fluor 546/647 or Cy3 can be used to label miRNAs. Fluorescently labelled miRNAs are hybridized to the microarray; this results in a specific binding of the labeled miRNAs to the corresponding probes (Figure 5). Detection of fluorescence emission from labelled miRNAs can be obtained at different positions on the glass slide; this allows the evaluation of miRNA relative quantities in the specific sample by analyzing the fluorescence signal intensity. In some microarray platforms expressions from two different samples can be compared by using two different fluorophores [112,131]. In the last years different variants have been proposed for detecting miRNAs with the microarray technology, with technical variations ranging from immobilization chemistry, probe design, sample labeling or microarray chip signal-detection methods [132]. Various commercial microarray platforms are available for miRNA detection and quantification, and their performances in measuring the differential expression of miRNAs have been evaluated. Several studies have compared different microarray platforms and have shown significant differences in miRNA quantification and biases in their ability to determine miRNA expression profiles (refs including [133,134]). The different capability of the platforms could be due to technical procedures such as the enzymatic reactions and amplification steps performed in the preparation of the RNA samples, or may arise from microarray probe design, microarray manufacturing, detection hardware, or algorithms for the extraction of intensity signals and subsequent data analyses. Moreover, a single microRNA microarray can contain from hundreds to thousands of probes, each with its own T_m_, but hybridization is usually carried out at one single temperature resulting in distortion in the fluorescent signal and hybridization biases [131]. Irrespective of the microarray platform used, the task of normalization in miRNA microarray experiments is dealt with by statistical normalization techniques [107,108]. However, for mRNA microarrays it is possible to identify several housekeeping genes, in the case of miRNA microarrays, similarly to qPCR (see above), it is hard to find noncoding RNAs that do not vary depending on the source of the sample. As microarrays contain probes for hundreds of noncoding RNAs at the same time, a solution to the problem is to use mathematical methods for the normalization. These methods (also used for mRNAs) rely on the assumptions that (1) only a small number of miRNAs are differentially expressed in samples under investigation versus control samples; (2) overexpressed miRNAs are roughly balanced by under-expressed miRNAs. Different techniques, including median, cyclic loess, quantile normalization, have been used in miRNA microarray experiments, with the latter being the most used technique and the one that works best in reducing differences in miRNA expression values for duplicate tissue samples [107]. In general, miRNA microarrays are best suited for an initial screening and do not possess sufficient sensitivity and specificity for absolute quantification.

### 3.4. Small RNA Sequencing

**Figure 6:**
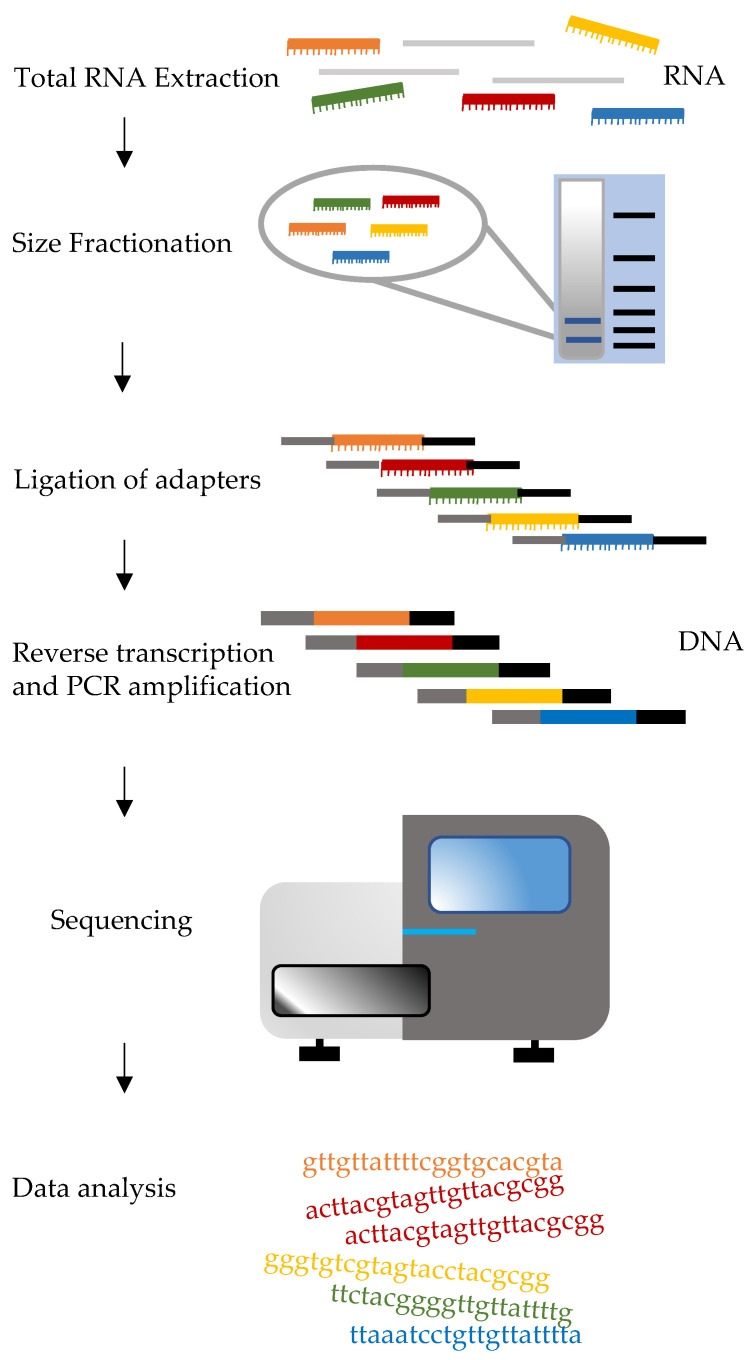
Diagram of small RNA Sequencing workflow. For the sake of simplicity, only five small RNA types are indicated (colored miRNAs). However, this technology has the potentiality to detect several thousands of different small RNA molecules, irrespective of whether they are known or they have never been described. Details of the experimental steps vary depending on the library preparation method and on the NGS platform used.

Recent advances in next-generation sequencing (NGS) have made miRNA profiling flexible and reliable. Small RNA sequencing (sRNA-Seq) involves different steps: Total RNA isolation, size fractionation, ligation of adapters at both 3′ and 5′ ends of small RNAs, reverse transcription and PCR amplification (Figure 6). At the end of the sequencing, results are represented by unique reads that are then mapped to the latest annotated genome or to sequences of mature miRNAs that can be found on miRbase (www.mirbase.org). Library construction can be performed using different kits, depending on the sequencing platforms being used. More specifically, available protocols and strategies for sRNA-Seq can be divided into two categories; strategies using adapters with invariant ends like TruSeq (Illumina), NEBNext (New England Biolabs) or CleanTag (Trilink Biotech) and strategies using adapters with four degenerate nucleotides at the ligation ends (4N adapters) like NEXTflex (Bioo Scientific). Adapter ligation steps may cause serious bias, known as “sequence bias”, due to RNA sequence/structure effects, resulting in the preferential ligation of certain sRNAs with a given adapter sequence, at the disadvantage of others. sRNA-Seq displays larger sequence bias when compared with long RNA-Seq [135]. Moreover, different 4N adapter protocols generated datasets of different quality, thereby suggesting that other parameters such as temperature and ligations times should be considered while performing sRNA-Seq [136]. Instead of modifying the adapters other groups tried to reduce bias through the optimization of reaction conditions by using a thermostable DNA/RNA ligase or by trying ligation at different temperatures [136].

Formation of side products such as adapter dimers is another source of bias for sRNA library preparation. To reduce this kind of bias, the available library preparation kits either use strategies such as purification steps to avoid excess 3′ adapter before 5′ adapter ligation, or the use of complementary oligonucleotides that inactivate the 3′ adapter [135]. A detailed sRNA-Seq protocol including RNA purification from mammalian tissues, library preparation, and raw data analysis is available here [137].

Small RNA-seq experiments are particularly impacted by the highly non-normal distribution of expression of different miRNAs, and scientists are aware of unanswered questions about the optimal approach for processing the data obtained [138]). Often, especially in biofluids, a few miRNAs account for a very large fraction of total reads while many other miRNAs contribute just a small percentage of reads. A number of normalization methods are available for sRNA-seq, falling in three categories: (1) Scaling; (2) normalizing to achieve similar data distributions; and (3) Reads Per Kilobase Mapped (RPKM). There appears to be a lack of consensus regarding the optimal normalization method, but it is common knowledge that different methods can result in different results in downstream analysis, particularly differential expression analysis (DE) [138,139,140].

## 4. Indirect Methods for miRNA Measurement: Reverse Transcription Only 

Most of the current methods for analysis of circulating miRNAs require RNA extraction and purification and small RNA enrichment. This often leads to partial loss of the c-miRNAs due to the incomplete denaturation of proteins and/or incomplete precipitation of RNA, which may impair detection of low abundance miRNAs. Also the reverse transcription step, required before the application of several measurement methods, such as qPCR or ddPCR, is known to affect target quantification [141]. Therefore, direct detection methods would be desirable. Skipping of the above mentioned steps would greatly shorten the time to obtain miRNA biomarker quantification and decrease the cost of circulating miRNAs measurement compared to traditional RNA-based assays. More importantly, direct quantification methods have the potential for yielding a more reliable quantification of circulating miRNAs compared to the approaches relying on RNA extraction.

In 2011 the group of David Hoon published a reverse-transcription quantitative real-time PCR (RT-qPCR) assay (RT-qPCR-DS) that was used for the first time for the direct detection of circulating miRNAs in serum [142]. The technique was tested on a cohort of 102 patients sera with different stages of breast cancer and 20 healthy female donors. This was the first work demonstrating the utility of an RT-qPCR-DS assay for c-miRNAs. By using this direct measurement method, they minimized mechanical errors, reduced duration of the measurement procedure and its costs. Hoon et al. were able to prove the diagnostic and prognostic potential of circulating miR-21 for detecting and staging breast cancer independently of estrogen-receptor status or age [142].

In 2013 Ho et al. reported an assay to quantify expression levels of mRNAs and miRNAs directly from cell lysates (cell to Ct), without RNA isolation [143]. In this work, optimization of five different commercial cell lysis buffers (Biorad, Qiagen, ABI, Roche and Signosis) and a custom buffer was performed in order to adapt them for the direct quantification of mRNAs and miRNAs from cell lysates (cell-to-Ct) by RT-qPCR. Moreover, they compared the TRIzol standard extraction method with the different cell lysis buffer mentioned above. Comparisons were based on the variability (measured as coefficient of variation, CV) and the efficiency of the extraction (measured as Ct values) of target mRNAs and miRNAs from human cell lines from different tissues. The work demonstrated that miRNA and mRNA quantification analysis using the custom and the Biorad commercial lysis buffer showed a comparable pre-analytical performance with the results obtained by conventional Trizol extraction. Thus, higher throughput, higher rapidity and accuracy of analysis were obtained by directly detecting different RNA species from the same cell lysate without the RNA isolation step.

In 2016 a direct quantification method for measuring plasma miRNAs contained in vesicles was reported by the Zhao group [144]. With this method, RNA isolation and purification steps are not required, since miRNAs-containing exosomes and/or microvesicles are chemically or heat denatured. miRNAs are released and miRNA-specific reverse transcription and quantitative real-time PCR amplification can be performed. This method was used for validating changes in circulating miR-106a levels in metastatic breast cancer patients. Blood samples were collected from breast cancer patients and healthy females of the same age. Plasma was then mixed with a Tris and EDTA based denaturing buffer and heated in a water bath at 75 °C for 5 min, then cooled on ice, and centrifuged for 10 min at 4 °C. The supernatant was used for miRNA reverse transcription and real-time PCR. This direct miRNA analysis provided evidence for the potential of miR-106a as a biomarker specifically for metastatic human breast cancer. By avoiding RNA extraction and purification, this method shortened the experimental time, decreased the cost for circulating miRNAs measurement compared to the traditional RNA-based assays and demonstrated to be more efficient and accurate for measuring circulating miRNAs compared to the RNA-based approaches.

Another direct measurement protocol applied on miRNA in plasma and serum was published in 2019. This method, named as “Direct S-Poly(T) Plus” relies on the denaturation of miRNA-protein complexes with proteinase K and a specific lysis buffer [145]. Since the composition of the lysis step is a key point to allow efficient release of miRNAs from protein complexes, six different combinations of buffers, enzymes and temperatures were tested. The best combination resulted to be the following: 20 μL 2× reaction buffer (50 mM Tris–HCl, 150 mM NaCl, 10 mM MgCl_2_, 1 mM ATP, 0.5 mM dNTP, pH 8.0) + 1 μL proteinase K + 20 μL plasma, 50 °C for 20 min, and then 95 °C for 5 min. The denaturation phase was followed by simultaneous polyadenylation and reverse transcription. The RT primer was composed of an oligo(dT)_11_ sequence and six miRNA-specific bases. This primer design ensured specificity and thermodynamic stability of the miRNA-primer hybrid. A hot-start Taq polymerase was then used for the amplification of cDNA. A volume of 20 μL plasma/serum allows detection of approximately 100 miRNAs in about 2 h. This approach demonstrated higher sensitivity compared with the RNA purification-based miRNA assays. Single miRNA detection was possible with plasma volumes as low as 0.0003 μL, compared to the inputs reported with the methods described by Asaga et al. (0.625 μL) and Zhao et al. (0.02 μL) [142,144]. Seven miRNAs (hsa-miR-423-5p, hsa-miR-451a, hsa-miR-30b-5p, hsa-miR-27b-3p, hsa-miR-199a-3p, hsa-let-7d-3p, and hsa-miR-423-5p) were identified as potential biomarkers in colorectal cancer using hsa-miR-93-5p as reference molecule. Moreover these miRNAs were able to discriminate stage I colorectal cancer from healthy subjects, underlining the feasibility of early diagnosis [145].

## 5. Indirect Methods for miRNA Measurement: RNA Extraction Only

### Nanostring’s nCounter Platform

**Figure 7:**
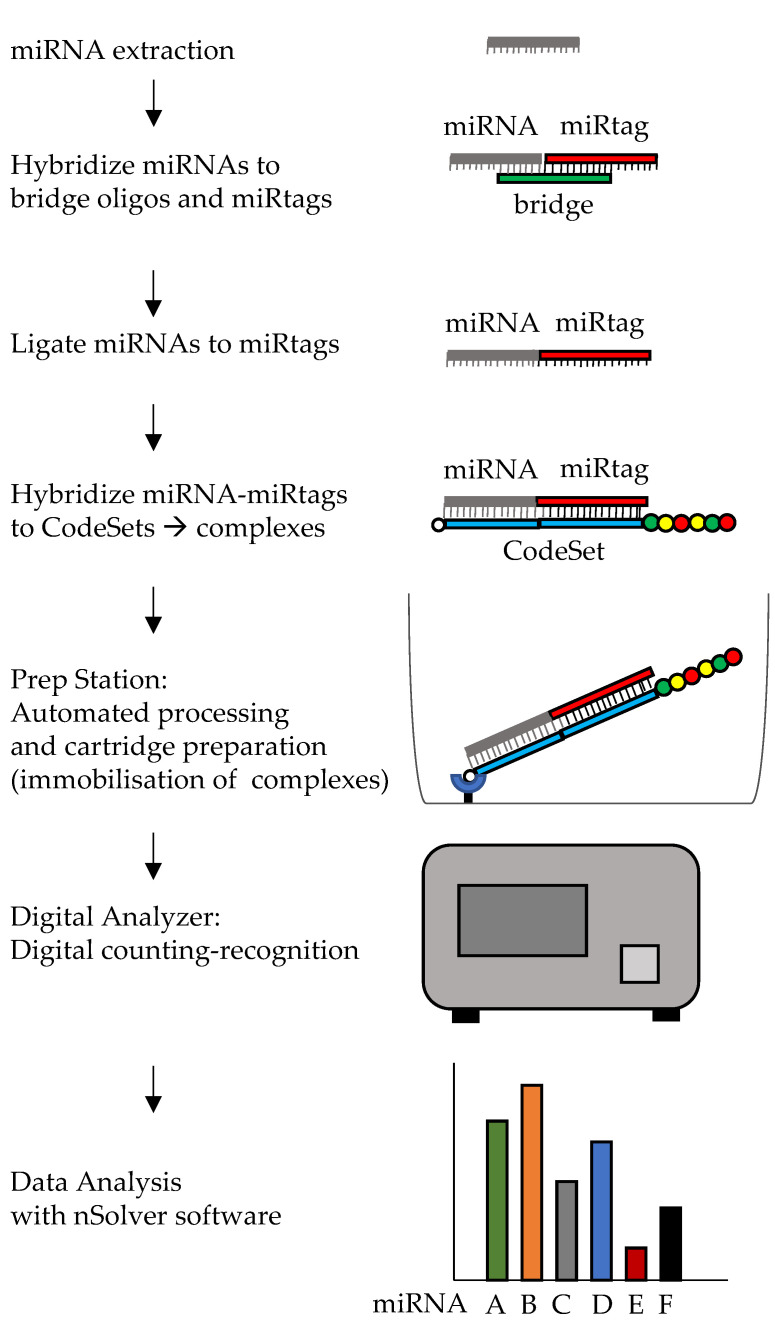
Diagram of Nanostring nCounter workflow. After hybridization with bridge oligos, miRNAs are ligated to miRTags and hybrized to capture and reporter probes (constituting the Codesets): the biotinylated capture probe will be used to immobilize the complexes on the cartridge in the Prep Station, whereas the reporter probes will allow identification and counting of each type of miRNA complex by the Digital Analyzer, by means of a unique code formed by a combination of fluorophores. Wash steps are not shown.

The nCounter platform by Nanostring relies on hybridization and can directly quantify miRNAs after their extraction from biological samples, without the need for reverse transcription or amplification. To allow for efficient detection of up to 800 miRNAs in a single analysis, given the short size and variable composition in bases of the different miRNAs, a strategy has been devised to make the hybridization temperature for the different targets uniform. This is obtained by the addition of “miRTags” (Figure 7). These oligonucleotides have a unique sequence for each miRNA to be detected, so that the different miRNA-miRTag sequences will have the same melting temperature, allowing for multiplexing. Indeed, by the addition of the miRTag, hybridization can occur at the same temperature, independently of the GC content of the specific miRNA. A bridge oligonucleotide is used, that hybridizes partly to the specific miRNA and partly to the unique miRTag oligonucleotide, to permit their ligation, after which the bridge oligonucleotide will be removed with a purification step. Finally, a target specific, biotinylated capture probe will serve for immobilization onto a streptavidin coated cartridge and a reporter probe will be used for recognition of the specific target being quantified, based on a barcode, a combination of 6 molecules of four different fluorophores. The digital analyzer will provide quantification for each of the targets measured by the assay. The assay includes several internal controls and probe sets for exogenous spike-ins eventually added by the users, for normalization purposes. Normalization can also be done by exploiting the multiplex nature of the analysis, as described in Section 2.3. When compared to HTG EdgeSeq and sRNA-seq, nCounter technology showed no bias for miRNA sequence and GC content and decreased susceptibility to cross-detection of miRNAs, but also a lower sensitivity [146]. Costs of instruments and turnaround time (about 2 days) may be obstacles to its widespread application in the clinic.

## 6. Direct Methods for miRNA Measurement from Biofluids

### 6.1. HTG EdgeSeq

**Figure 8:**
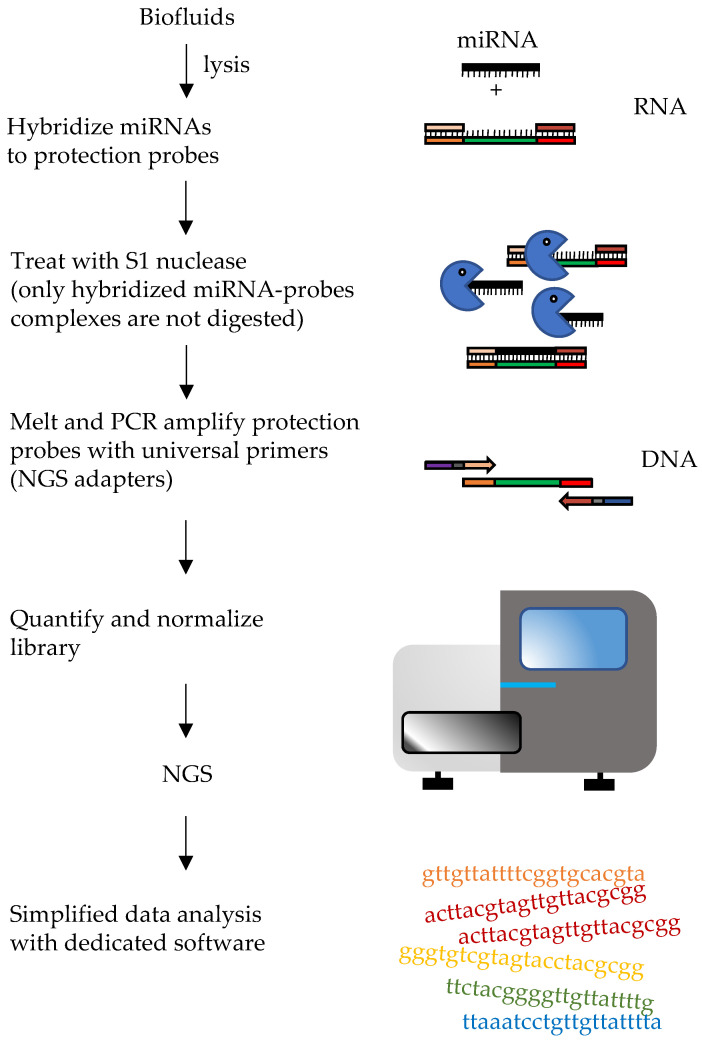
Diagram of HTG Edgeseq workflow. The hybridization, S1 nuclease treatment and PCR steps are depicted. These steps allow it to protect probes bound to target miRNAs, removing other RNAs and unbound probes. Wash steps are not shown.

In 2018 an approach called HTG EdgeSeq, developed by HTG Molecular Diagnostic, Inc. (Tucson, AZ, USA), was described for the direct screening of plasma c-miRNAs. By eliminating RNA extraction and reverse transcription, it simplified both library and sample preparation for RNA and miRNA targeted sequencing. Probes are used to form double stranded molecules with targets and protect the complexes from degradation with S1 nuclease. The probes also carry “wings”, extensions that will allow introduction of adapters for NGS by PCR, protected by “wingmen”. Single stranded nucleic acids (targets and unbound probes), will be degraded, leaving the target-probe complexes available for subsequent steps (Figure 8). This approach allows direct quantification of transcripts or miRNAs, allowing to increase the throughput and to considerably reduce bioinformatic analyses and time to results (~36 h). Profiles of more than 2000 miRNA transcripts can be performed starting from a relatively small amount of initial sample. Normalization can be performed as classical sRNA-Seq. Moreover, the HTG EdgeSeq costs are consistent with other next-generation sequencing (NGS) approaches. In a study by Songia and coworkers peripheral blood from four healthy subjects was collected. Samples were barcoded, purified and quantified using a KAPA Library Quantification kit [147]. Overall HTG-sequencing with Illumina NextSeq was performed leading to the evaluation of the expression of 2083 miRNAs starting from just 15 μL of plasma. In addition, the study compared the performances of techniques that are commonly used for miRNA detection like quantitative PCR (qPCR) and the chip-based digital PCR (dPCR) and a strong correlation between qPCR and dPCR with HTG was found [147]. In another comparative study, the performance of HTG Edgeseq was compared with that of classical RNA-seq, Fireplex and Nanostring nCounter platforms in measurement of synthetic miRNA pools or miRNAs from plasma samples [146]. HTG Edgeseq showed the smallest coefficient of variation when repeated measures were performed and low bias in measuring “ratiometric pools” of synthetic miRNAs. Moreover, it could detect variation in the levels of placenta-specific miRNAs between pregnant and non-pregnant women [146]. 

### 6.2. Branched DNA

**Figure 9:**
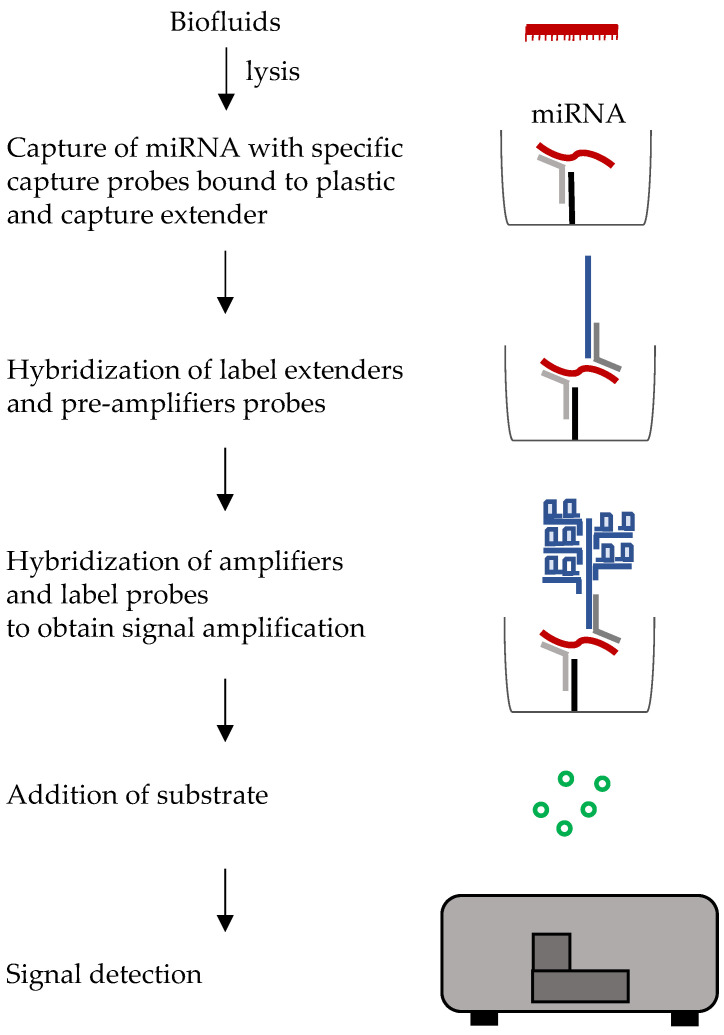
Diagram of standard branched DNA workflow. A cascade of hybridization steps, using different types of probes, leads to signal amplification.

Branched DNA is a technique widely used in virology for detection and quantitation of viral genomes. It relies on hybridization and signal amplification rather than target amplification. Capture oligonucleotides are immobilized on a support and are complementary to an extender sequence. The latter also hybridizes to the target nucleic acid. Signal amplification is obtained by adding further label extender, preamplifier and amplifier oligonucleotides. The latter will be conjugated with an enzyme, i.e., alkaline phosphatase or horseradish peroxidase and detection and quantification is obtained after adding the appropriate substrate (Figure 9). This technique does not require RNA extraction but can detect the molecules of interest directly in biofluids. However, the short length of miRNAs may represent an obstacle for efficient quantification. Recently, an ultrasensitive method for microRNA detection using a branched DNA based SERS (surface-enhanced Raman scattering) platform was proposed. Enhancement of signals up to 10^11^ was obtained by coupled surface plasmons at nanoscale junctions between noble metal nanoparticles, in this case silver nanoparticle films, that allowed multiplexing and direct detection of miRNAs and other macromolecules, including proteins, directly in biofluids. This novel platform was shown to have a limit of detection of 10 attomolar for miR-223 [148]. The first step to evaluate the performance of the bDNA assay, is to separate the variability of replicate measurements in the assay, from the biological variability. This technique requires specific expertise and may suffer of low reproducibility.

### 6.3. Multiplex Circulating Fireplex

**Figure 10:**
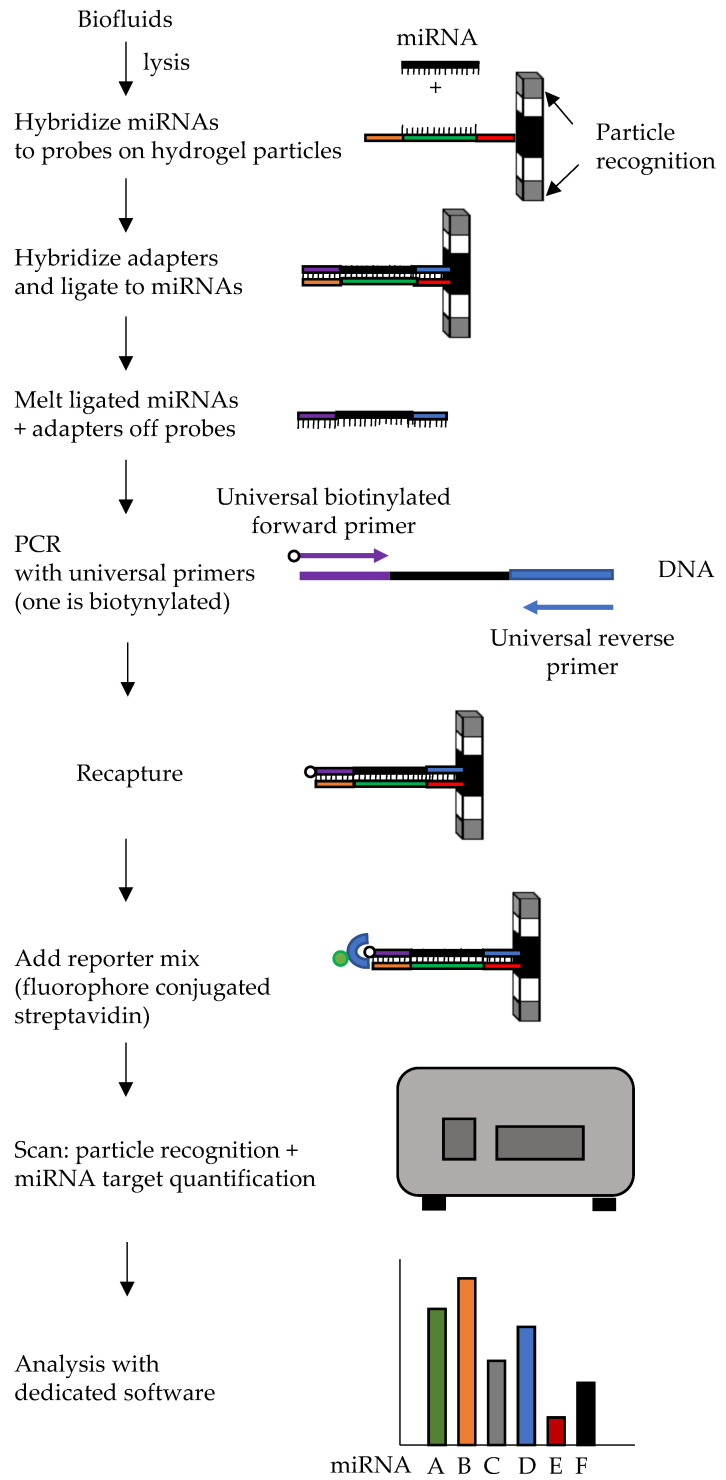
Diagram of Fireplex workflow. The hydrogel particle carries a combination of fluorophores at the two ends, for particle recognition by flow cytometry, and probes for miRNA capture and adapters ligation. For the sake of simplicity, only one probe is shown, but several identical probes are present on each particle, to bind a specific miRNA. The sequence hybridizing to the target miRNA is shown in green, the sequences hybridizing to the universal adapters that will be ligated to the miRNA are in orange and red. In each well particles for up to 65 different targets are present. Wash steps are not shown.

The Multiplex Circulating Fireplex miRNA assay by Abcam works in a 96 well format, allowing simultaneous detection and quantification of up to 65 different miRNAs in a single well, and analysis of 96 samples at a time [149]. The method is based on the use of hydrogel particles, composed of different functional parts (Figure 10). A central region, carrying probes complementary to each specific miRNA to be detected, is surrounded by two terminal regions with differing fluorescent intensities that function as barcodes to identify the specific miRNA recognized by the particle. miRNAs are captured by hybridization to the central region and ligated to universal adapters. They are then eluted and undergo PCR amplification by using biotinylated primers complementary to the adapters. After PCR, the biotinylated miRNAs are recaptured on the hydrogel nanoparticles. Fluorescence is measured on a flow cytometer or by fluorescence microscopy: Fluorophores conjugated streptavidin, bound to biotin, is used for quantification of miRNAs, whereas identification of the target being measured relies on detection of the fluorescent combination and intensity in the hydrogel nanoparticle. Normalization can be obtained using one or more of the miRNAs included in the assay, or by using the geometric average of all detected targets.

An advantage of this method is that it allows quantification directly from biofluids, starting from 10 μL, without the need for miRNA extraction or processing. The Fireplex platform has been recently applied for miRNA biomarker studies in different diseases, including psoriatic arthritis, Duchenne muscular dystrophy and cardiac disease, although an extensive literature is still lacking due to the novelty of the technique [150,151,152]. In the oncologic field, Leng et al. showed a good concordance of measures obtained with Fireplex, performed directly on plasma samples, compared to the measures obtained by qPCR, upon quantification of 11 specific miRNAs as putative lung cancer biomarkers [153]. The Authors showed that the two methods had equivalent analytic performance, e.g., similar precision and repeatability. Another advantage of Fireplex was the shorter times from sample to results. On the other hand, in a comparative study analyzing Edgeseq, small RNA seq, Fireplex, and nCounter platform performances, Godoy et al. showed that although it had comparable sensitivity and specificity, Fireplex had higher Coefficient of variation compared to the other technologies [146].

### 6.4. Chem-NAT

**Figure 11:**
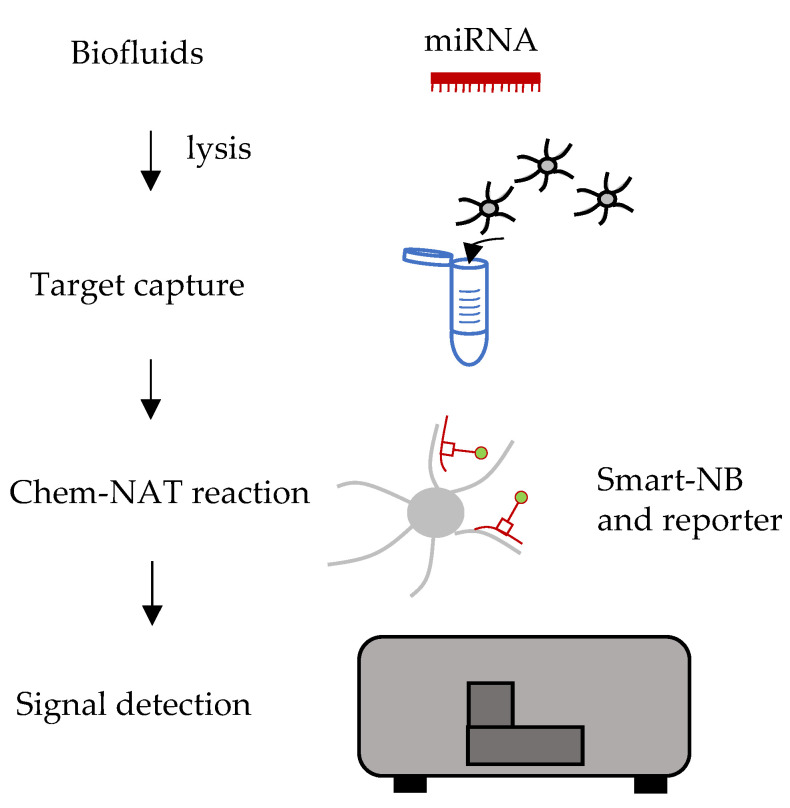
Diagram of Chem-NAT workflow. Chem-NAT allows direct detection of the analyte of interest. First, the probes (in the example coupled to magnetic beads) are injected directly in the sample of interest together with a proper lysis buffer. Second, the Chem-NAT reaction occurs, by specific incorporation of the SMART-NB modified with a reporter. Finally, the signal produced by the reporter is detected.

DestiNA Genomics patented an innovative chemical method that can be applied for nucleic acid detection and quantification (Chem-NAT) [154]. The chemistry relies on peptide nucleic acid (PNA) probes complementary to the target of interest and aldehyde-modified nucleobases (SMART-NB). The PNA probes contain an abasic site that reacts with the SMART-NB creating a covalent bond (Figure 11).

Chem-NAT quantitation relies on two main events: (1) The hybridization of the PNA probe to the target and (2) the specific incorporation of the SMART-NB which follows the standard Watson–Crick base pairing rules via dynamic chemistry. Only the correct SMART-NB is favored and eventually sufficiently stable to enter the abasic site of the PNA probe. This mechanism allows for (1) single-base specific resolution and (2) labeling of the PNA probe. The detection may be done with any methodology (chemiluminescent, fluorescent, or colorimetric), depending on the type of modification the SMART-NB carries. The technology may be used for direct detection from several sources, including biofluids, avoiding RNA extraction, RT and/or PCR amplification and it has been validated in several contexts for miRNAs analysis [155,156,157,158,159].

Chem-NAT draws most of its advantages by being a direct detection assay. It does not need any normalization step, since, by means of a calibration curve with known concentrations of a synthetic version of the analyte, an absolute quantification in the sample of interest is possible. Table 2 summarizes the duration, throughput and multiplexing capabilities of the techniques described in this review.

## 7. Conclusions

A comprehensive knowledge of c-miRNAs and their origin, as well as of other classes of non-coding RNAs including lncRNAs and circular-RNAs, has still to be obtained. The combination of miRNAs with other biomarkers should be evaluated. However, a clinical diagnostic test requires not only specific analytes that can identify the presence of a condition, be it cancer or other diseases, but also a reliable method for their quantification. The present review describes up to date techniques that can be applied, and whose performance can be compared, to address the issue of reliable miRNA biomarker quantification. With stringent guidelines, a detailed description of the procedures used for miRNA quantification in published papers and increased application of direct measurement methods, we expect that a step closer to their clinical application can be reached. Independent validation studies are required before microRNA measurement can be translated into clinical applications.

## Figures and Tables

**Table 1:** Summary of the different technical limitations during miRNA quantification.

Phase of the Analysis	Challenges
Pre-analytical phase	− Gender, smoking status and physiological conditions (diet, active exercise, circadian rhythm) − Comorbidities − Type of sample (plasma, serum, saliva, urine, or FFPE and flash-frozen tissues) − Modality of sample collection (e.g., venipuncture) or time from collection to processing − For plasma: Which type of anticoagulant − Sample processing (centrifugations steps) − Sample storage
Analytical phase	− RNA extraction-different kits − Primer design: Difficulties to discriminate pri-miRNA, pre-miRNA and mature forms − Reverse transcription: Specific primers or universal primers could introduce amplification biases − Extraction/quantification efficiency affected by miRNA sequence composition
Post-analytical phase (normalization)	− Single reference molecule − Set of reference molecules − Mathematical normalization − Different software for the selection of the best reference molecule (Normfinder, geNorm, Bestkeeper) − Calibration curve

**Table 2:** Summary of the different time, throughput and multiplexing of the different methods described in this review.

		Method	Time	Throughput	Multiplexing
INDIRECT MEASUREMENTS	RNA extraction and RT	qPCR	<6 h	Medium	Low
		digital PCR	<6 h	Medium	Low
		Microarray	~2 days	Low	High
		NGS	1–2 weeks	Medium	High
	RT only	RT-qPCR-DS	<4 h	Medium	Low
		Cell to Ct RT-qPCR			
		Direct S-Poly(T) Plus	<4 h	Medium	Low
		RT-qPCR	<4 h	Medium	Low
	RNA extraction only	Nanostring Platform	~2 days	Low	High
DIRECT MEASUREMENTS		HTG EdgeSeq	~36 h	Medium	High
		bDNA	~12 h	Low	High
		Fireplex	~6 h	High	Medium
		Chem-NAT	~3 h	Medium	Low

## Abbreviations

RNA	ribonucleic acid
ncRNAs	non coding RNAs
lncRNAs	long non coding RNAs
tRNAs	transfer RNAs
5S rRNA	5S ribosomal RNA
siRNAs	silencing RNAs
piRNAs	piwi RNAs
snRNAs	small nucleolar RNAs
miRNAs	microRNAs
nt	nucleotides
RISC	RNA-induced silencing complex
TGF-β	transforming growth factor beta
Ago2	argonaute protein 2
HDL	high density lipoprotein
NPM1	nucleophosmin 1
DROSHA-DGCR8	drosha-DiGeorge syndrome chromosomal region 8
3′UTR	3′untranslated region
5′UTR	5′untranslated region
EV’s	extracellular vesicles
FFPE	formalin Fixed Paraffin Embedded
c-miRNAs	circulating microRNAs
ADC	adenocarcinoma
SCC	squamous cell carcinoma
RT-qPCR	reverse-transcription quantitative PCR
RT	reverse transcription
LNA	locked nucleic acid
ddPCR	digital droplet PCR
Cy3	cyanine 3
Tm	melting temperature
NGS	next generation sequencing
sRNA-Seq	small RNA sequencing
lRNA-Seq	long RNA sequencing
FAM	fluorescein
SERS	surface-enhanced Raman scattering
PNA	peptide nucleic acid
SMART-NB	aldehyde-modified nucleobases
